# Prognostic prediction models for psoriatic arthritis in psoriasis: A systematic review

**DOI:** 10.1111/jdv.70561

**Published:** 2026-06-18

**Authors:** Fatimah Khoda, Yasmin Khan, William W. Y. Haw, Sayeda Nahar, Claire Hodkinson, Hector Chinoy, Richard B. Warren, Glen P. Martin, Zenas Z. N. Yiu

**Affiliations:** ^1^ The Dermatology Centre, Salford Royal Hospital, Northern Care Alliance NHS Foundation Trust Manchester Academic Health Science Centre Salford UK; ^2^ Division of Musculoskeletal and Dermatological Sciences, School of Biological Sciences, Faculty of Biology, Medicine and Health The University of Manchester Manchester UK; ^3^ Department of Dermatology St Mary's Community Health Campus Portsmouth UK; ^4^ West Hertfordshire Teaching Hospitals NHS Trust Watford UK; ^5^ The University of Manchester Library University of Manchester Manchester UK; ^6^ Department of Rheumatology Salford Royal Hospital, Northern Care Alliance NHS Foundation Trust, Manchester Academic Health Science Centre Salford UK; ^7^ Division of Informatics, Imaging and Data Science, Faculty of Biology, Medicine and Health University of Manchester Manchester UK

**Keywords:** medical dermatology, prognostic prediction models, psoriasis, psoriatic arthritis, risk

## Abstract

**Background:**

Psoriatic arthritis is a chronic inflammatory arthropathy that affects up to 22% of individuals with psoriasis. Identifying patients at higher risk of developing this disease presents a window of opportunity for dermatologists to consider treatments that can limit its progression.

**Objectives:**

This study aims to systematically identify and evaluate the predictive performance, risk of bias and reporting quality of prognostic prediction models developed to estimate the risk of psoriatic arthritis in patients with psoriasis.

**Method:**

A systematic search was conducted across Medline, Embase, Cochrane, Web of Science, pre‐print servers and grey literature, with the search completed on 15 October 2025. Data extraction was guided by the Checklist for Critical Appraisal and Data Extraction for Systematic Reviews for Prediction Modelling Studies (CHARMS). The Prediction model Risk of Bias Assessment Tool (PROBAST) and the Transparent Reporting of a multivariable prediction model for Individual Prognosis Or Diagnosis (TRIPOD‐AI) were used to evaluate risk of bias and reporting, respectively.

**Results:**

Fifteen studies were included after full‐text screening, comprising of 490 models incorporating a variety of predictors. The most frequently used predictors were demographic variables (age, gender, BMI), genetic markers (HLA‐B27, HLA‐B08, HLA‐C*06), clinician and patient‐reported outcomes (PASI, DLQI), clinical features and lifestyle factors. Nine studies used machine learning methods, such as Bayesian probabilistic methods and random forest, whilst the remainder used traditional statistical approaches such as regression and risk scores. The C‐statistic ranged from 0.53 to 0.99. Calibration, where reported, was modest and sensitivity and specificity were reported in seven studies, often with inconsistent thresholds and reporting. Only three studies performed external validation, which reduced discriminative performance, raising concerns about generalizability.

**Conclusions:**

Future studies should incorporate a variety of predictors to develop comprehensive models that reflect the aetiology of Psoriatic arthritis whilst ensuring adequate reporting and validation in external cohorts to allow assessment of model performance.

## Graphical abstract

This systematic review evaluated prognostic prediction models used to assess the risk of future psoriatic arthritis in psoriasis patients. The included studies reported modest to moderate performance measures, with limited external validation, inadequate reporting and high risk of bias.

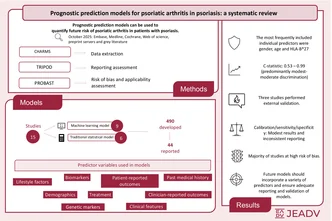

**Why was the study undertaken?**

- Several risk factors are associated with developing psoriatic arthritis in patients with psoriasis.
- Prognostic prediction models can be used to quantify the future risk of developing disease.
- We aimed to review prognostic prediction models used to detect psoriatic arthritis in patients with psoriasis.

**What does this study add?**

- Predictor variables used include patient demographics, clinical features, patient and clinician‐reported outcomes, past medical history, family history, treatments, lifestyle factors, biomarkers and genetic markers.
- Model discrimination ability was variable with a C‐statistic of between 0.53–0.99.
- Key issues identified include poor reporting and high risk of bias.

**What are the implications of this study for disease understanding and/or clinical care?**

- This review highlights that current prediction models are not yet suitable for clinical use, often due to the use of limited clinical datasets and poor reporting quality.
- Future research should focus on external validation of existing models alongside developing models using diverse populations using a variety of predictor variables with adequate reporting to limit the risk of bias.

## INTRODUCTION

Psoriatic arthritis (PsA) is an autoimmune, inflammatory arthropathy associated with psoriasis and characterized by debilitating arthralgia, joint stiffness and dactylitis. Despite its reported prevalence of 14–22%,^1^ a significant proportion of cases remain undiagnosed.^2^ PsA also has significant systemic implications, increasing the risk of metabolic syndrome, cardiovascular disease and mental health disorders.^3^

Early identification of PsA can prevent irreversible joint damage, lowering both disease burden and healthcare costs.^4^, ^5^ Inflammatory pathways in the synovial and entheseal tissues gain momentum long before clinical symptoms appear,^6^ presenting an opportunity to implement treatment strategies that may halt, delay or even prevent the development of PsA. Dermatologists are ideally positioned to identify patients, as those with severe psoriasis, an independent risk factor for PsA, are more commonly treated in secondary care. The use of targeted biologic treatments, such as interleukin (IL)‐23, IL‐12/23, tumour necrosis factor‐alpha and IL‐17 inhibitors, has been shown to reduce PsA risk.^7^

Whilst individual risk factors can help identify patients at increased risk of psoriatic arthritis, their clinical application is limited, as clinicians must subjectively estimate the impact of each factor, potentially introducing bias. Prediction models provide a more objective and data‐driven approach by combining multiple variables to calculate the probability of disease development.^8^ These models, also known as risk scores, prognostic indices or prognostic prediction models, use statistical methods to predict the likelihood of a future outcome occurring over a specified period.

There is currently no review that summarizes the performance and clinical relevance of models in predicting PsA risk in psoriasis, making it unclear whether any are suitable for clinical use. This systematic review aims to identify and critically appraise prognostic prediction models in psoriasis patients, to summarize their predictive performance and evaluate their methodological quality and risk of bias.

## MATERIALS AND METHODS

The systematic review was reported in accordance with PRISMA guidelines. A protocol was registered on PROSPERO (CRD42022299479) prior to the initiation of the study.

### Outcomes

The study's primary outcome is the predictive performance of prognostic prediction models for PsA in patients with psoriasis. Secondary outcomes included identifying predictors used in multivariable models, evaluating bias and reporting quality.

### Eligibility criteria

The inclusion criteria covered studies evaluating prognostic prediction models developed for use following a diagnosis of cutaneous psoriasis.

Studies evaluating both adults and/or children were considered. Papers primarily focusing on model validation, whether internal or external, and studies examining the impact of prediction models were also included. Only models incorporating more than one variable were eligible. All modelling techniques, such as regression, and machine learning approaches, were included. If multiple models were discussed within a single article, all models were evaluated.

Studies that identified predictors or risk factors without including a multivariable model were excluded. Diagnostic prediction models, defined as models designed to detect the presence of a condition, and screening tools were excluded.

If multiple publications of the same model existed, only one paper was included, prioritized in the following order: peer‐reviewed publication, pre‐peer‐reviewed publication and conference abstracts. Case reports and papers not written in English were also excluded.

### Data sources and search strategy

MEDLINE, EMBASE, Web of Science, the Cochrane Library and the pre‐print servers (BioRxiv, MedRxiv and Xriv) were searched on 15 October 2025. Grey literature was searched using Scopus and conference indices. Reference lists of eligible studies and identified systematic reviews were screened to check for additional relevant articles. The search strategy was finalized with input from an information specialist (CH) and is found in Table S1.

### Screening and article selection

Four independent reviewers (FK, YK, WH, SN) screened the titles and abstracts to determine eligibility using Rayyan software^9^ using a blinding tool. The reviewers conducted independent full‐text analyses for articles classified as ‘included’ or ‘maybe’. If conflicts remained, they were initially discussed among the reviewers (FK, YK, WH), with an independent reviewer (ZY) consulted if no resolution was reached.

### Data extraction

Three standardized tools ensured a systematic approach to data extraction and quality assessment. The Critical Appraisal and Data Extraction for Systematic Reviews of Prediction Modelling Studies (CHARMS) checklist was used to guide data extraction,^10^ the Prediction model Risk of Bias Assessment Tool (PROBAST)^11^ was used to assess risk of bias and the Transparent Reporting of a Multivariable Prediction Model for Individual Prognosis or Diagnosis Initiative plus Artificial Intelligence (TRIPOD + AI)^12^ statement was used to assess reporting adequacy. Two reviewers (FK, YK) carried out data extraction and appraisal independently, utilizing a standardized data extraction table. RBW, who was a co‐author on two of the included studies, was not involved in the screening, extraction or appraisal process of this review.

### Statistical analysis of models

From each model, statistical analysis of the predictive performance was completed. Specifically, we extracted the reported discrimination using the C‐statistic (also known as the area under the curve [AUC]), sensitivity and specificity at any selected cut‐off points. Where reported, we extracted calibration plots and summary measurements, including the calibration intercept (calibration‐in‐the‐large) or calibration slope. We extracted estimates of performance at both the development and validation datasets, where applicable.

For models with multiple external validation studies, a meta‐analysis using RevMan was considered for the outcomes mentioned above. Additionally, a separate meta‐analysis was planned to explore sources of heterogeneity across ethnicity, country, timing for when the models should be used and psoriasis subtypes.

## RESULTS

### Study selection

The search, conducted on 15 October 2025, identified 4425 publications (Figure 1), of which 30 underwent full‐text review. Ten studies were excluded as the models were diagnostic.^13^, ^14^, ^15^, ^16^, ^17^, ^18^, ^19^, ^20^, ^21^, ^22^ Two conference abstracts were excluded due to overlap with peer‐reviewed publications of the same study.^23^, ^24^ One review paper^25^ a research programme report,^26^ and one additional study were excluded as the outcome was risk of developing arthritis mutilans in PsA patients.^27^ Fifteen studies were included in the systematic review.^28^, ^29^, ^30^, ^31^, ^32^, ^33^, ^34^, ^35^, ^36^, ^37^, ^38^, ^39^, ^40^, ^41^, ^42^ 490 models were developed; 44 models were reported (Table 1).

**FIGURE 1:**
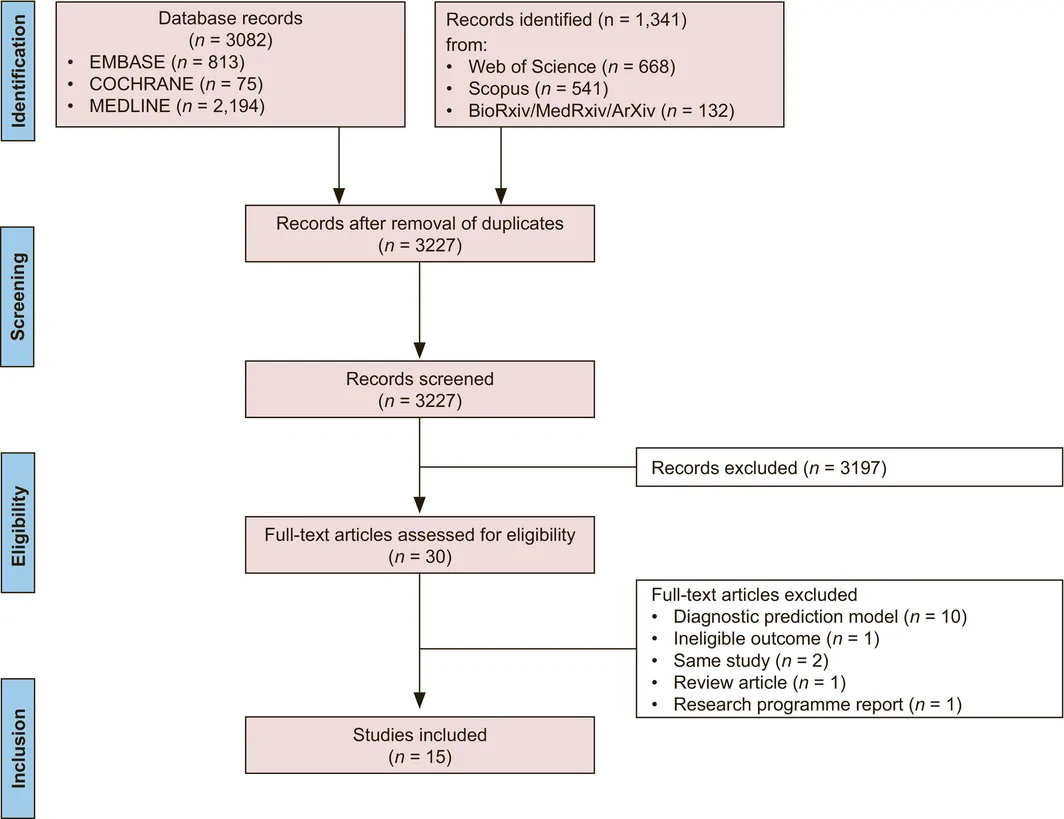
PRISMA 2020 flow diagram.

**TABLE 1:** Summary of highest performing prediction models per study based on the c‐statistic.

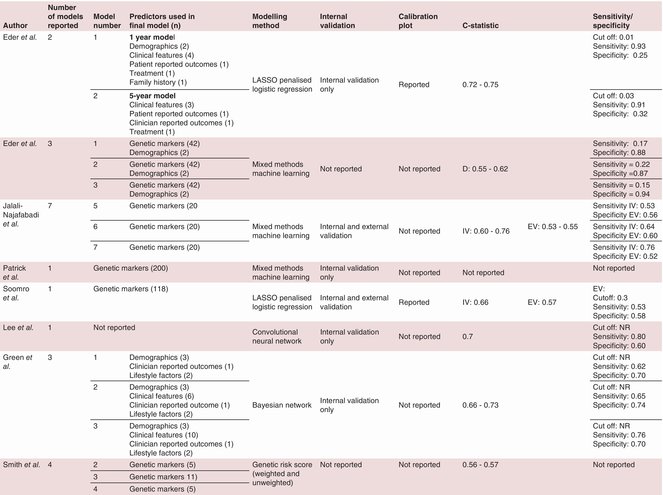
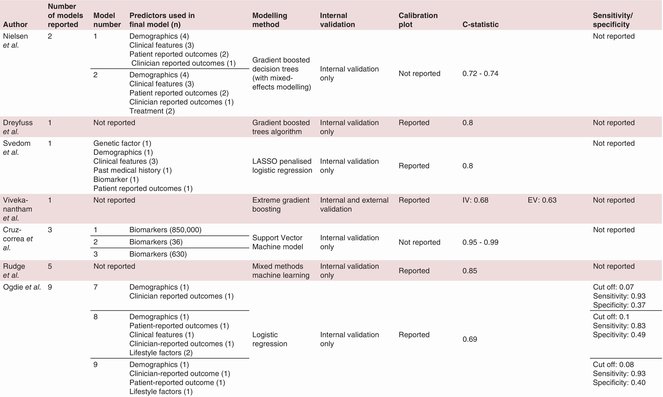

*Note*: Three highest performing models per study (based on C‐statistic) are presented. Internal validation refers to evaluation of the model performance in the same dataset as model development but using statistical methods such as cross‐validation and bootstrapping. External validation refers to evaluation of model performance in an independent dataset, ideally drawn from the target population in whom the model is intended for use.

Abbreviations: D, development dataset; EV, external validation; IV, internal validation.

### Description of predictors

The number of predictors used varied substantially, ranging between 2 and 850,000, with biomarker and genetic marker models typically incorporating larger predictor sets (see Table S2). A range of predictor variables was used (Figure 2). Demographic predictors were the most frequently used predictor category, specifically gender and age, as shown in Table 2, which summarizes predictor frequency, restricted to those utilized in ≥2 studies. Genetic markers were also frequently included. Three studies used single nucleotide polymorphisms (SNPs),^29^, ^31^, ^32^ one used human leukocyte antigens (HLA) alleles,^30^ one used a combination of SNPs and HLA alleles,^35^ and one study included HLA‐B27 alongside other clinical predictors.^38^ Among studies reporting the exact genetic markers used, these were predominantly related to pathways involved in antigen presentation (HLA class I), IL‐17/Th17 signalling and skin barrier function.

**FIGURE 2:**
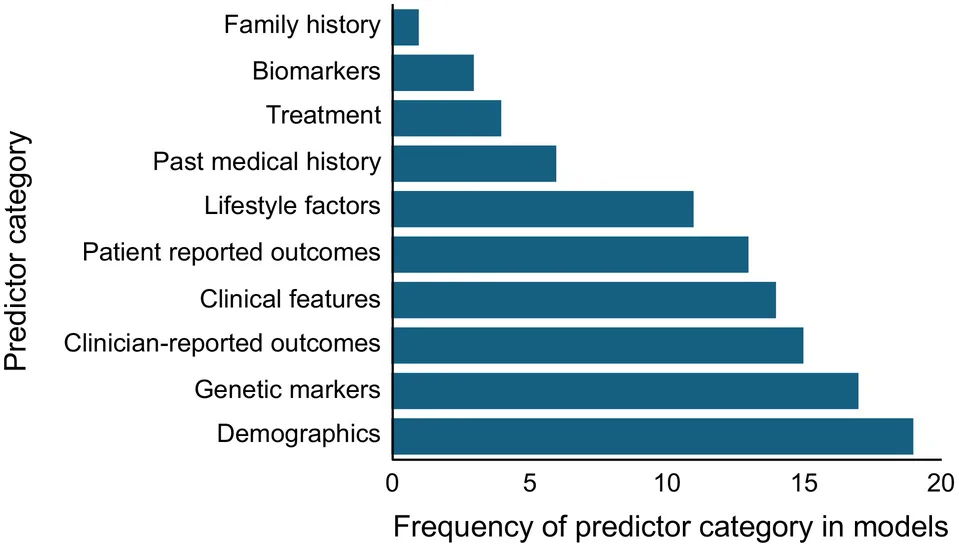
Frequency of predictor categories used across all reported models. Individual predictor weights were not consistently reported and therefore could not be included.

**TABLE 2:** Frequency and reported associations of predictors included in prognostic prediction models across studies (predictors identified in ≥2 studies).

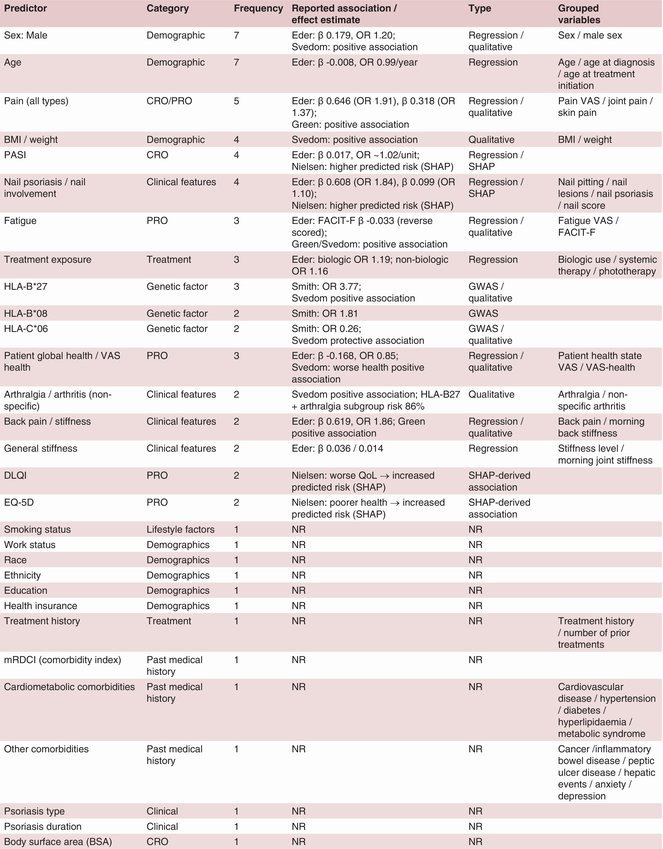
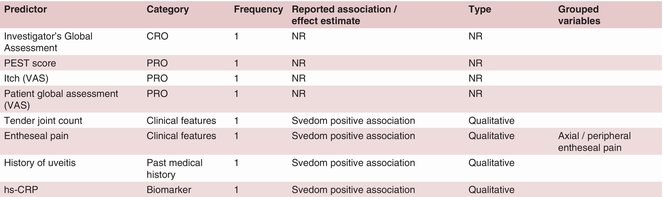

*Note*: Frequency is defined as the number of studies in which the predictor was included in the final model. Frequency of predictors does not reflect the predictive value of the individual predictors in PsA, as most models did not assess the size of the effect of the predictor on the outcome. Where available, reported effect estimates and direction of association are shown as presented in the original studies.

Abbreviations: BMI, Body Mass Index; CRO, clinical reported outcomes; DLQI, Dermatology Life Quality Index; EQ‐5D, EuroQol 5 Dimension; HLA, human leucocyte antigen; PASI, Psoriasis Area and Severity Index; PRO, patient‐reported outcomes; VAS, Visual Analogue Scale.

Clinical features were predominantly musculoskeletal and examination‐based variables (15 models) such as arthralgia, tender and swollen joint count, enthesitis, morning stiffness and axial symptoms. Clinician‐reported outcomes were included in 10 models, most commonly Psoriasis Area and Severity Index (PASI), whilst patient‐reported outcomes were incorporated in 13 models including visual analogue scale (VAS) pain score and Psoriasis Epidemiology Screening Tool (PEST). Lifestyle factors such as smoking and alcohol were utilized in 11 models. Four studies did not report on the number or type of predictors used in the final model.^23^, ^33^, ^37^, ^39^

### Modelling method

The studies employed a range of modelling methods (Table 1). Nine studies used machine learning approaches. Bayesian probabilistic methods such as Naïve Bayes variants and Bayesian networks were commonly used, alongside other machine learning methods (Table 1). Several studies applied multiple machine learning approaches to the same dataset, which impacted predictive performance.^29^, ^30^, ^41^ Patrick et al.^31^ developed an initial diagnostic model, subsequently using Bayesian risk estimation to convert classifier scores into individual PsA risk probabilities.

Eder et al.,^28^ Soomro et al.,^43^ Svedom et al.^38^ and Ogdie et al.^42^ used traditional statistical regression. Conversely, Eder et al.^29^ and Jalali‐Najafabedi et al.^30^ both used logistic regression within a machine learning framework, utilizing automated variable selection to improve model performance.

Jalali‐Najafabedi et al.^30^ used seven machine learning methods to generate up to 448 initial models. The best‐performing model from each method was validated using the Biomarkers and Stratification to Optimize outcomes in Psoriasis (BSTOP) GWAS dataset, a UK‐based study that collected tissue samples from over 7000 patients with psoriasis and external validation using UK Biobank data.^44^ Soomro et al.^32^ also used BSTOP and UK biobank datasets for internal and external validation, respectively.

Three studies drew their samples from the University of Toronto Psoriasis Cohort, a prospective study designed to identify risk factors associated with psoriatic arthritis in psoriasis patients.^28^, ^29^, ^40^

### Model performance and validation

Cruz‐correa et al.^40^ developed three models using DNA methylation markers. The highest discriminative performance (0.99) was achieved using 630 methylation markers identified after adjusting for age, sex, BMI and smoking status.^40^ The addition of clinical predictors did not improve performance and was therefore omitted.

Rudge et al.^41^ produced five models with good discrimination; however, calibration was acceptable in only two models. Dreyfuss et al. and Svedom et al. also reported good discriminative ability, with C‐statistics of 0.80 (Figure 3), but calibration was limited, and sensitivity or specificity were not reported.^37^, ^38^

**FIGURE 3:**
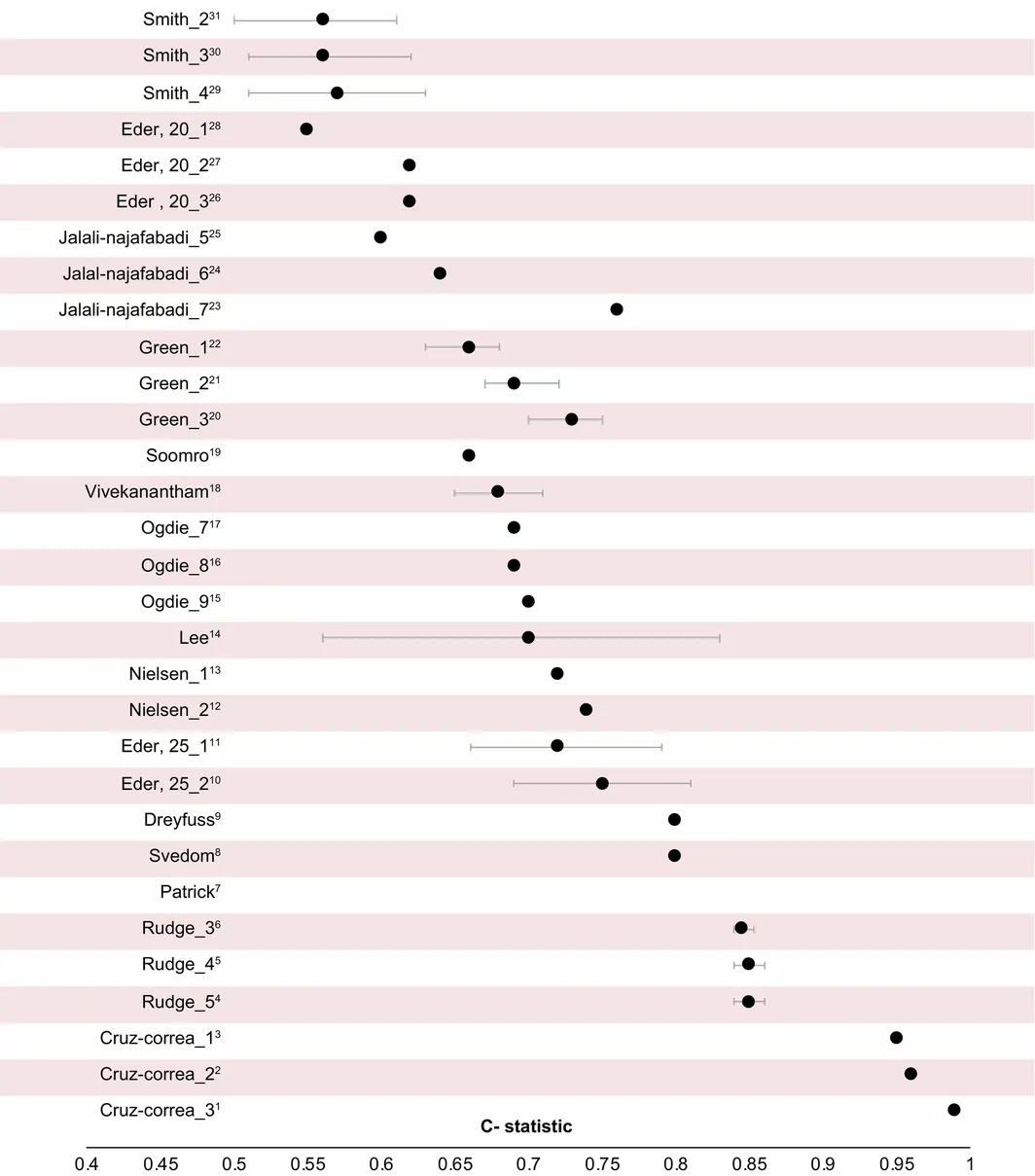
Forest plot of internal validation C‐statistic (AUC) for the best‐performing models. Confidence intervals are shown where reported.

The PRESTO study^28^ developed 1‐ and 5‐year clinical predictor prediction models, with a C‐statistic of 0.72 and 0.75, respectively. A web‐based calculator, with risk tables for specific patient scenarios, has been published online.^28^ External validation was not performed. Eder et al.^29^ additionally developed a genetic marker model, which performed poorly in comparison (Figure 3).

Using a developmental dataset only, Smith et al.^35^ developed four models, using either 11 genetic loci or 5 HLA allotypes. Both weighted and unweighted analyses were performed, which had limited impact on predictive ability^35^ (Figure 3).

Soomro et al.^32^ developed a model using 118 single nucleotide polymorphisms (SNPs). The model's discriminative ability dropped from 0.66 to 0.57 in external validation. A calibration slope was reported as 1.41, indicating overfitting and poor generalizability.

The best‐performing Jalali‐Najafabadi model was KNNC (K‐Nearest Neighbour Classifier), achieving a C‐statistic of 0.76,^30^ however, performance declined to 0.53 on external validation. A reduction in C‐statistic in external validation was also observed in the model produced by Vivekanantham et al.^39^ dropping from 0.68 to 0.63.

Green et al.'s^34^ best‐performing model incorporated musculoskeletal symptoms associated with PsA. The model achieved a C‐statistic of 0.73 (95% CI: 0.70–0.75) and acceptable sensitivity and specificity (Table 1), but with no classification threshold.

Lee et al.^33^ developed a model using Taiwanese registry data, achieving a c‐statistic of 0.70 (95% CI: 0.559–0.833), with acceptable sensitivity, modest specificity; however, no classification threshold was provided (Table 1).

The inclusion of medication/treatment use as variables in Nielsen et al.'s^36^ second model had minimal impact on its discriminative ability, with a marginal increase in the c‐statistic.

Patrick et al.^31^ did not report on discrimination or calibration for their prognostic prediction model.

Ogdie et al.^42^ reported on discrimination, calibration, sensitivity and specificity with defined thresholds for all three models developed; however, overall performance was modest.

A meta‐analysis was not completed due to significant heterogeneity in the datasets, including inconsistent reporting, differences in the types of predictor variables and variation in definition and measurement.

### Model outcome

The primary outcome across all studies was the risk of developing PsA. However, outcome measurement methods varied and included CASPAR criteria, clinical record codes, expert opinion.

### Risk of bias and reporting

Risk of bias was low in participant selection for the majority of studies, besides Dreyfuss et al., Svedom et al. and Vivekanantham et al. where it was unclear primarily due to limited reporting associated with conference abstract publication (Table 3).^37^, ^38^, ^39^

**TABLE 3:** Risk of bias and applicability assessment of prognostic prediction models using PROBAST.

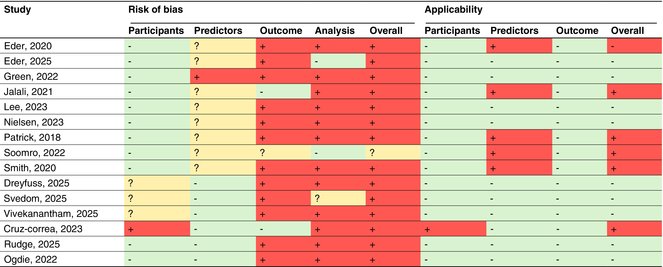

*Note*: Green ‘–’ = low risk of bias/low concern of applicability, red ‘+’ = high risk of bias/high concern for applicability, yellow ‘?’ = unclear risk of bias/unclear risk of applicability.

Concerns with the outcome domain arose as several studies did not use a standardized or validated diagnostic framework to define PsA development.

In the analysis domain, insufficient reporting of missing data handling and predictor weights increased the risk of bias. In addition, reporting on key performance measures such as calibration, sensitivity, specificity, confidence intervals and assessment of overfitting through external validation was frequently missing.

Applicability, defined as alignment of participants, predictors and outcomes with the intended clinical use of the model, was of high concern in five genetic marker model studies as the markers are unlikely to be readily available in routine clinical practice.

Inadequate reporting was widespread, particularly in sample selection, sample size justification, blinding, analytical methods, data/code sharing, model specification, model performance, patient and public involvement and usability in clinical practice. Both risk of bias and applicability concern was high in Cruz et al.^40^ as the 1:1 matched nested case–control study design may distort outcome prevalence and baseline risk.

## DISCUSSION

This systematic review summarized the performance of prognostic prediction models for PsA in patients with psoriasis. A substantial number of models have been reported, using a broad range of modelling methods and predictor variables. Although many models demonstrated good performance internally, in cases where external validation was undertaken, model performance declined. Therefore, their generalizability and performance in independent, representative populations remain uncertain.

Additionally, poor reporting and methodological limitations introduced a risk of bias, further limiting clinical applicability. Calibration, where assessed, was modest, which raises concern regarding real world performance.^45^ Misestimation of risk, particularly underestimation, may delay identification of high‐risk patients and subsequent management. Heterogeneity in outcome definitions, timing, the use of homogenous populations for model development and large predictor sets likely contribute to these findings.

Cruz‐correa et al.^40^ achieved excellent discrimination using DNA methylation markers, however, risk of bias and concern for applicability were high. Nonetheless, the study prospectively identified markers associated with ‘converters’ (patients who developed PsA) compared with non‐converters. These differing methylation patterns may reflect pre‐clinical disease activity in those who later develop PsA. A key feature of these biomarkers is their dynamic nature, which may better capture the differences between the two groups over more fixed clinical or genetic predictor variables.

However, predictor variability may reduce generalizability, and calibration is vital to ensure predicted risk reflects observed risk in the target population.

No specific modelling method appeared consistently to improve performance, a finding consistent with existing literature.^46^

Among the five studies using primarily genetic predictors, the C‐statistic ranged from 0.53 to 0.76, indicating poor to modest discriminative performance. Polygenic risk scores require large, diverse datasets to ensure reliability. Only Patrick et al.^31^ reported study population ethnicity, identifying a European bias. Additionally, all studies except that by Lee et al.^33^ which was conducted in Taiwan, were based in Europe or North America. Similar limitations related to statistical power and ethnic bias have been reported in skin cancer polygenic scores.^47^ Mitigation strategies, such as recalibration and the inclusion of diverse study samples, are essential to ensure that predicted risk matches observed outcomes in clinical practice. A further challenge is the substantial genetic overlap between psoriasis and PsA; ongoing research aims to identify such markers, which may improve future model performance.^43^

Eder et al.^29^ included demographic variables with genetic predictors, though these had minimal impact on performance. Integration of broader clinical and patient‐reported variables may improve predictive accuracy and better reflect the complex aetiology of PsA^48^ including psychosocial factors such as stress, fatigue and mood that may also influence the disease pathway.^49^, ^50^

Most studies relied on existing data sources not specifically created for model development or validation, raising concerns about missing data, variability in data collection and selection bias, which limit generalizability and reliability. In contrast, Eder et al.,^28^ Svedom et al.^38^ and Cruz‐correa et al.^40^ developed their modes using prospective cohort data, which may have contributed to higher discrimination performance observed in these studies.

Patients with severe psoriasis may receive biologic treatment, which could effectively ‘treat’ pre‐clinical PsA, potentially affecting phenotype discrimination and model performance. in these patients. Subgroup analysis for biologic‐naive patients versus those on treatment may clarify this effect. Nielsen et al.^36^ included treatment/medication as a predictor and did not significantly affect performance.

Only Eder et al.^28^ commented on the practical considerations of implementing models in clinical practice. Key issues include time, resource implications, as well as the perspectives of key stakeholders such as patients and clinicians. Impact studies and decision curve analysis can estimate a clinical benefit threshold by balancing the risk of developing PsA against the possible adverse effects of treatment.

The risk of bias was of high concern in all studies except Soomro et al.,^32^ primarily due to insufficient reporting on key domains such as blinding, the absence of a standardized outcome definition and limited assessment of model performance. Poor reporting is a well‐recognized issue in modelling studies.^51^, ^52^, ^53^ Without transparent reporting of model development and performance, clinicians are unable to judge model applicability to their target population.

Future work should aim to externally validate models of high performing models is needed to assess generalizability in other cohorts and mitigate bias through clear reporting in line with TRIPOD + AI guidelines and careful study design, including clearly defined outcomes, blinding and transparent model development. A reproducible code should be made publicly accessible on repositories such as GitHub.

This systematic review has several strengths. We explored an area with potential to significantly impact outcomes for patients with PsA. A robust search strategy was implemented, covering multiple key medical databases as per PRISMA guidelines, as well as grey literature to minimize publication bias. Additionally, a protocol for this systematic review was registered with PROSPERO, in line with Cochrane guidance.

However, there are some limitations. Whilst we searched Google Scholar, we could not manually screen all retrieved articles due to the limited number of screeners.

## CONCLUSIONS

In summary, this systematic review identified several prognostic prediction models for PsA in psoriasis patients, most of which demonstrated variable performance, ranging from excellent to poor and used a variety of predictors. Biomarkers and models with a variety of predictors performed better than solely genetic marker models. There was inconsistency in the reporting of key performance measures, introducing a high risk of bias and causing difficulty assessing clinical utility.

Further research should focus on externally validating models that demonstrate good discrimination and low risk of bias and consider recalibrating them for use in relevant populations. There is also a need to develop better reported models, ideally incorporating demographic and clinical predictors to improve both performance and clinical applicability.

## AUTHOR CONTRIBUTIONS

**Fatimah Khoda –** search strategy formulation, screening, data extraction, appraisal, writing – original draft preparation, review and editing, **Yasmin Khan –** screening, data extraction, appraisal, review and editing, **William Haw** – screening, review and editing, **Sayeda Nahar** – screening, review and editing, **Claire Hodkinson** – search strategy, review and editing, **Hector Chinoy –** review and editing, supervision, **Richard Warren** – review and editing, supervision, **Glen Martin** – review and editing, supervision, methodology, **Zenas Yiu –** methodology, review and editing, conceptualization, supervision.

## FUNDING INFORMATION

This research was supported by the National Institute for Health and Care Research Manchester Biomedical Research Centre (award NIHR203308). ZZNY is supported by an MRC Clinician Scientist Fellowship (grant ref.: MR/Z504026/1).

## CONFLICT OF INTEREST STATEMENT

The authors declared no conflicts of interest.

## ETHICAL APPROVAL

Ethical approval was not required for this study as it was a systematic review of published literature and did not analyse any individual patient data.

## ETHICS STATEMENT

Not Applicable.

## DISCLAIMER

The views and opinions expressed therein are those of the authors and do not necessarily reflect those of the National Institute for Health and Care Research, National Health Service or Department of Health.

## Supporting information


**Table S1.** Search strings used to search literature.

**Table S2.** Summary of studies included, using the CHARMS checklist. CI, confidence intervals; D, developmental data set; EV, external validation; IV, internal validation; NR, not reported; PPV, positive predictive value.

**Data S2.** PRISMA 2020 checklist.

## Data Availability

The data that support the findings of this study are available from the corresponding author upon reasonable request.
