# Supplementary material for: Prognostic prediction models for psoriatic arthritis in psoriasis: A systematic review
Source: J Eur Acad Dermatol Venereol. 2026 Jun 18;40(11):1796–807. doi: 10.1111/jdv.70561 (PMC13695784; doi:10.1111/jdv.70561)
Supplement: Supplementary file 2 [file JDV-40-1796-s002.docx]

**Supplementary material**

Supplementary methods

The CHARMS checklist focuses on the following key areas: study design, participants, outcomes, candidate predictors, sample size, missing data, model development, performance, evaluation, results, and interpretation.

PROBAST evaluates four main domains: participants, predictors, outcomes, and analysis. Signalling questions guide the assessment of bias, with each domain rated as low, high, or unclear risk of bias.

For the search strategy, a combination of the terms including ‘Psoriasis’, ‘Psoriatic arthritis’, ‘Predict*’, ‘Model*’, and ‘Risk*’ were used alongside Boolean operators AND/OR to develop the search strategy (Supplementary table 1). The Ingui filter, designed to enhance sensitivity in searching for prediction literature in MEDLINE, was applied^54^. This filter has a reported sensitivity of 78% in identifying prediction literature^55^.

| **Database** | **Search Terms** |
| --- | --- |
| **MEDLINE** | Psoriasis AND psoriatic arthritis AND (Validat$ OR Predict$.ti. OR Rule$) OR (Predict$ AND (Outcome$ OR Risk$ OR Model$)) OR ((History OR Variable$ OR Criteria OR Scor$ OR Characteristic$ OR Finding$ OR Factor$) AND (Predict$ OR Model$ OR Decision$ OR Identif$ OR Prognos$)) OR (Decision$ AND (Model$ OR Clinical$ OR Logistic Models/)) OR (Prognostic AND (History OR Variable$ OR Criteria OR Scor$ OR Characteristic$ OR Finding$ OR Factor$ OR Model$)) |
| **EMBASE** | Psoriasis AND psoriatic arthritis AND (risk OR predict*) AND model |
| **Cochrane Library** | Psoriasis AND psoriatic arthritis AND (risk OR predict*) AND model* |
| **Web of Science** | Psoriasis AND psoriatic arthritis AND (risk OR predict*) AND model |
| **BioRxiv/MedRvix/ARxiv** | Psoriasis AND psoriatic arthritis AND (risk OR predict*) AND model |
| Scopus | Psoriasis AND psoriatic arthritis AND (risk OR predict*) AND model* |

**Supplementary Table S1**: Search strings used to search literature

Supplementary table S2

**Supplementary Table S2**: Summary of studies included, using the CHARMS checklist. D = developmental data set, IV = internal validation, EV = external validation, CI = confidence intervals, NR = not reported, PPV = positive predictive value,

Supplementary figure S1
